# Quantifying the contribution of direct runoff and baseflow to nitrogen loading in the Western Lake Erie Basins

**DOI:** 10.1038/s41598-022-12740-1

**Published:** 2022-06-02

**Authors:** Jung-Hun Song, Younggu Her, Tian Guo

**Affiliations:** 1 Agricultural and Biological Engineering Department & Tropical Research and Education Center, Institute of Food and Agricultural Sciences, University of Florida, Homestead, FL 33031 USA; 2 Agricultural and Biological Engineering Department, Purdue University, West Lafayette, IN 47907 USA

**Keywords:** Hydrology, Engineering

## Abstract

Soluble nitrogen is highly mobile in soil and susceptible to leaching. It is important to identify nitrogen transport pathways so that the sources can be efficiently targeted in environment management. This study quantified the contribution of direct runoff and baseflow to nitrate + nitrite loading by separating flow and nitrate + nitrite concentration measurements into two periods depending on whether only baseflow was present or not using baseflow separation methods. When both direct runoff and baseflow were present in streamflow, their nitrate + nitrite concentrations were assumed based on the hydrological reasoning that baseflow does not change rapidly, and streamflow mostly consists of direct runoff within a rainfall event. For this study, we obtained and investigated daily flow and nitrate + nitrite concentration observations made at the outlets of 22 watersheds located in the Western Lake Erie area. Results showed that baseflow was responsible for 26 to 77% of the nitrate + nitrite loads. The relative nitrate + nitrite load contributions of direct runoff and baseflow substantially varied with the sizes of drainage areas and agricultural land uses. Increases in drainage areas tend to prolong the travel time of surface runoff and thus help its reinfiltration into soil, which then could increase the baseflow contribution. In addition, the artificial drainage networks common in the agricultural fields of the study areas would promote the drainage of nutrient-laden excess water from soils. Such findings suggest the need for environmental management customized considering nitrogen transport pathways.

## Introduction

Quantifying loads of nutrients, including nitrogen and phosphorus, and identifying their transport paths are critical when developing management plans to protect the designated uses of downstream water bodies. Nutrient loads are generated and carried in the two principal forms, particulate and soluble. Most particulate nutrients move with direct runoff along flow paths on the ground surface. Thus, it is relatively easy to control its transport with conservation practices such as terraces and filter strips^1–4^. On the other hand, soluble nutrients are highly mobile, making it is difficult to identify their sources and transport routes^5^. Studies showed that the appropriate treatment of soluble nutrients is necessary for water quality improvement, especially in agricultural areas where intensive farming activities are taking place^1,6–9^.

As a part of the Corn Belt, the Western Lake Erie basin plays a vital role in providing land resources for food and energy production. However, intensive crop production systems have been identified as the primary sources of excessive nutrient loads resulting in water quality problems such as eutrophication and hypoxia of Lake Erie^1,10–15^. Tile drainage practices implemented to quickly drain excess soil water from agricultural fields may accelerate soluble nutrient loading to downstream water bodies including Lake Erie and make nutrient loading paths be more complicated to be identified clearly^1,16–19^. The contribution of tile drainage and baseflow to nutrient loading to Lake Erie needs to be quantified to develop targeted agricultural conservation practices for improved water quality of the lake.

Studies investigated how nitrogen can be loaded through different transport pathways using regression and watershed models^20,21^. Miller et al.^21^ linearly related the baseflow index (BFI) to the nitrate concentrations of winter streamflow to approximate the nitrate loading contribution of groundwater and runoff. They found that a significant portion of the annual nitrate loads were attributed to groundwater discharge. Liang et al.^20^ calibrated a Soil and Water Assessment Tool (SWAT) to stream discharge measurements made at the outlet of a study watershed to investigate the annual variations of groundwater and runoff contributions to total nitrate loads. Their study demonstrated that groundwater is the dominant pathway of nitrate loading throughout the year, regardless of seasons. However, it is still unclear how nitrogen loading is associated with watershed characteristics and how the contributions of baseflow and direct runoff may change depending on land uses and watershed management. In addition, the previous studies did not clearly isolate baseflow from direct runoff. For instance, baseflow was separated from direct runoff by applying an arbitrary threshold of 90% to streamflow^22^ or by implicitly assuming winter stream flows consist primarily of groundwater^21^. In addition, the nitrate concentrations of direct runoff and baseflow were assumed to be constant for the simplicity of analysis^21^. Such limitations might bring biases in the estimates of nitrogen loading contribution.

In this study, we quantified the contributions of direct runoff and baseflow to the loading of soluble nitrogen, nitrate + nitrite, to downstream water bodies using baseflow separation methods and hydrological reasoning about the temporal variations of baseflow’s nutrient concentrations and streamflow composition during a rainfall event. We collected and analyzed daily flow and nitrate + nitrite concentration measurements made at the outlets of 22 study watersheds, which have different land cover compositions draining water and nutrients to a downstream waterbody, to see the relationship between nitrogen loading pathways and watershed features.

## Material and method

### Study areas and data

**Figure 1:**
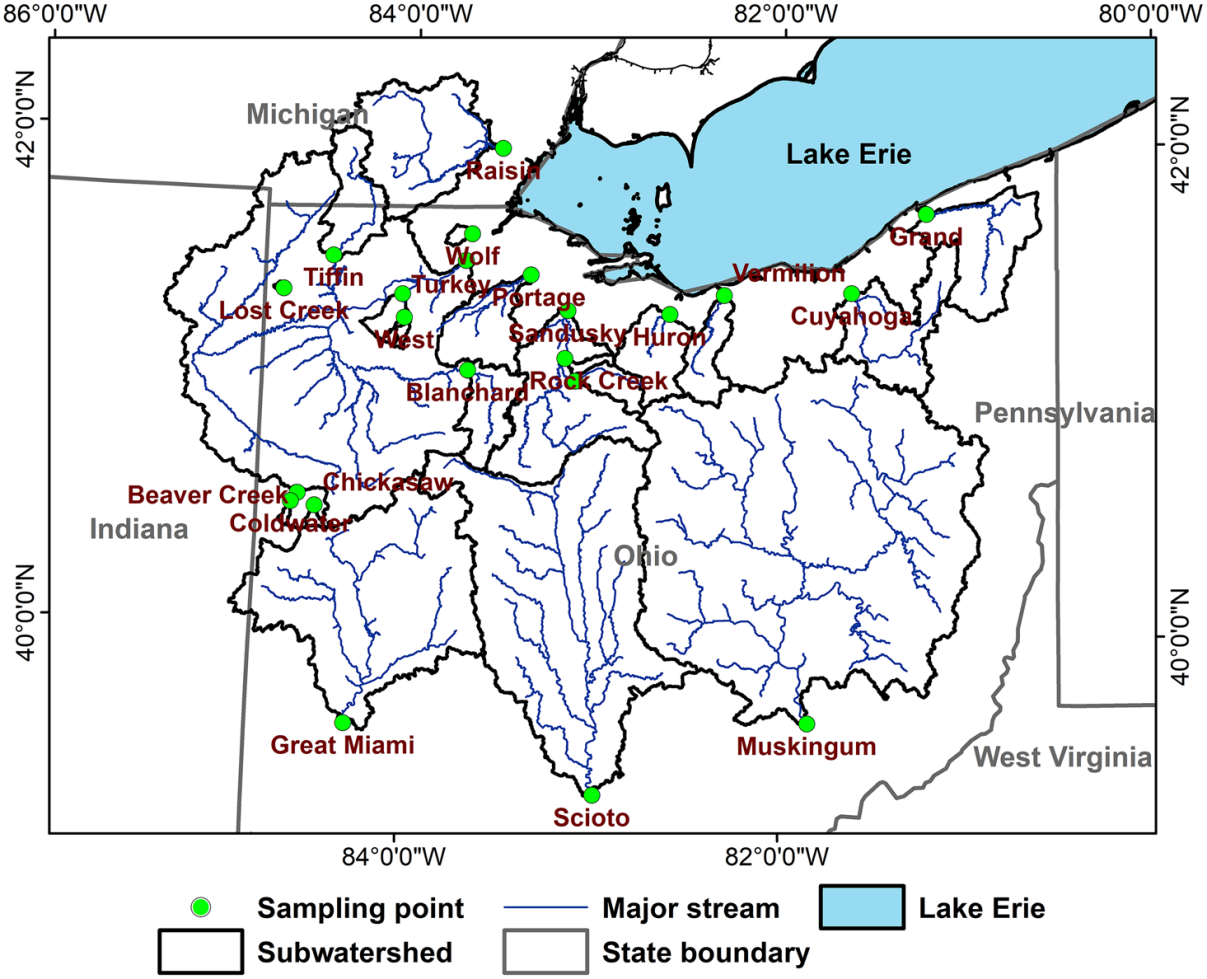
Locations of the National Center for Water Quality Research (NCWQR) water quality monitoring sites and associated watersheds draining water and nutrients to Lake Erie. (Figure was created by the authors using ArcMap 10.5: https://support.esri.com/en/products/desktop/arcgis-desktop/arcmap/10-5).

Lake Erie is one of the water bodies that have been suffering water quality issues such as harmful algal blooms (HABs) and hypoxia due to excessive nutrient loading from large agricultural fields (part of the corn belt) distributed in its drainage areas (Fig. 1 and Table 1)^7,16,23–25^. The West Lake Erie basin is known as the largest source of nutrients transported to the lake in particulate and dissolved forms^26,27^. Due to poorly drained soils, tile drainage systems have been widely used in the West Lake Erie basin^19,28^, and they can reduce the amount of direct runoff generated at fields by promoting infiltration and subsurface water movement^2,29^. However, studies showed that tile drainage systems could accelerate the loss of soluble nutrients from crop (corn, soybean, and winter wheat) fields and then may increase nutrient loading to Lake Erie^16,19,30^.

**Table 1:** NCWQR water quality monitoring periods and the watershed characteristics (NCWQR: https://ncwqr.org/monitoring/data/).

Watershed	Area (km^2^)	Monitoring period		Agriculture (%)	Pasture (%)	Forest (%)	Urban (%)	Other (%)	PDSC^a^ (%)	DD^b^ (km^−1^)
		Begin	End							
Beaver Creek	294	11/11/13	12/19/16	69.1	7.3	2.4	1.6	18.7	67.7	0.28
Blanchard	896	9/3/07	7/4/20	78.8	3.5	6.3	10.5	1.0	84.5	0.27
Chickasaw	43	10/24/08	7/4/20	79.0	8.9	2.8	9.1	0.1	75.9	0.50
Coldwater	30	10/15/12	7/1/20	68.8	14.0	2.6	12.2	2.5	79.6	0.43
Cuyahoga	1830	11/4/81	3/15/20	9.0	11.8	33.6	39.5	6.1	38.0	0.28
Grand	1774	3/1/88	8/10/06	40.0	–*	50.1	0.9	13.1	82.6	0.42
Great Miami	7019	4/22/96	7/4/20	64.5	8.5	8.6	17.0	1.4	54.6	0.33
Honey Creek	386	1/28/76	7/4/20	81.1	2.0	9.5	6.7	0.7	86.6	0.18
Huron	961	1/26/18	7/6/20	66.2	5.9	17.7	9.0	1.2	62.9	0.31
Lost Creek	11	11/12/07	7/4/20	77.5	8.6	7.9	4.3	1.8	63.8	0.69
Maumee	16,388	1/10/75	7/4/20	73.3	6.3	6.5	10.6	3.2	80.6	0.30
Muskingum	19,215	4/11/94	7/4/20	23.6	18.8	43.0	12.4	2.2	17.7	0.32
Portage	1108	8/30/10	7/4/20	84.4	1.3	4.5	9.0	0.8	85.0	0.19
Raisin	2698	3/6/82	3/16/20	49.6	18.7	11.0	10.8	10.0	61.3	0.42
Rock Creek	90	1/18/83	7/4/20	71.9	7.8	11.4	8.8	0.2	77.2	0.41
Sandusky	3239	12/7/74	9/30/04	77.6	4.3	8.8	8.1	1.2	80.3	0.37
Scioto	9965	4/23/96	7/4/20	61.7	8.6	10.9	17.3	1.5	63.4	0.35
Tiffin	1061	4/23/08	7/4/20	60.5	14.8	8.9	7.5	8.3	59.7	0.36
Turkey	300	4/30/18	7/6/20	89.5	1.0	3.0	6.3	0.2	98.7	0.43
Vermilion	679	11/12/00	7/21/08	72.8	–^c^	25.4	1.0	0.8	67.3	0.27
West	40	4/30/18	7/1/20	92.3	0.6	2.0	5.0	0.1	99.3	0.39
Wolf	64	9/24/18	7/6/20	12.0	7.5	28.8	48.1	3.6	65.6	0.20

^a^PDSC refers to Poor Drainage Soil Class (i.e., poorly drained, somewhat poorly drained, and very poorly drained) found in the USDA Soil Survey Geographic (SSURGO) dataset.

^b^DD refers to Drainage Density calculated using the USGS National Hydrography Dataset.

^c^Pasture is not separated from agriculture.

The NCWQR at Heidelberg University has been monitoring nutrient concentrations at Ohio and Michigan rivers conveying water and nutrients to Lake Erie on a daily basis since the mid-1970s^26,27,31,32^. The water quality data are open to the public and available at https://ncwqr.org/monitoring/data/. All 22 monitoring stations identified from the NCWQR database were included in this study (Table 1). The 22 study watersheds drain areas of 11 to 19,215 km^2^, and agricultural land uses cover 9.0 to 92.3% of the drainage areas. Annual precipitation depths vary from 833 to 1135 mm across the study watersheds in the Corn Belt areas. On average, 55% of annual precipitation fell in March to July^33^.

The water quality monitoring stations are located close to the USGS flow gaging stations so that nutrient concentrations can be converted to the loads. Such detailed water quality data provided critical information allowing the accurate identification of nutrient sources and transport pathways^26,27,31^.

The rank-based non-parametric Mann–Kendall (MK) statistical test^34,35^ was conducted to detect the significance of trends in the time series of the annual nitrate + nitrite load and flow/load contribution^36^.

### Load estimation

**Figure 2:**
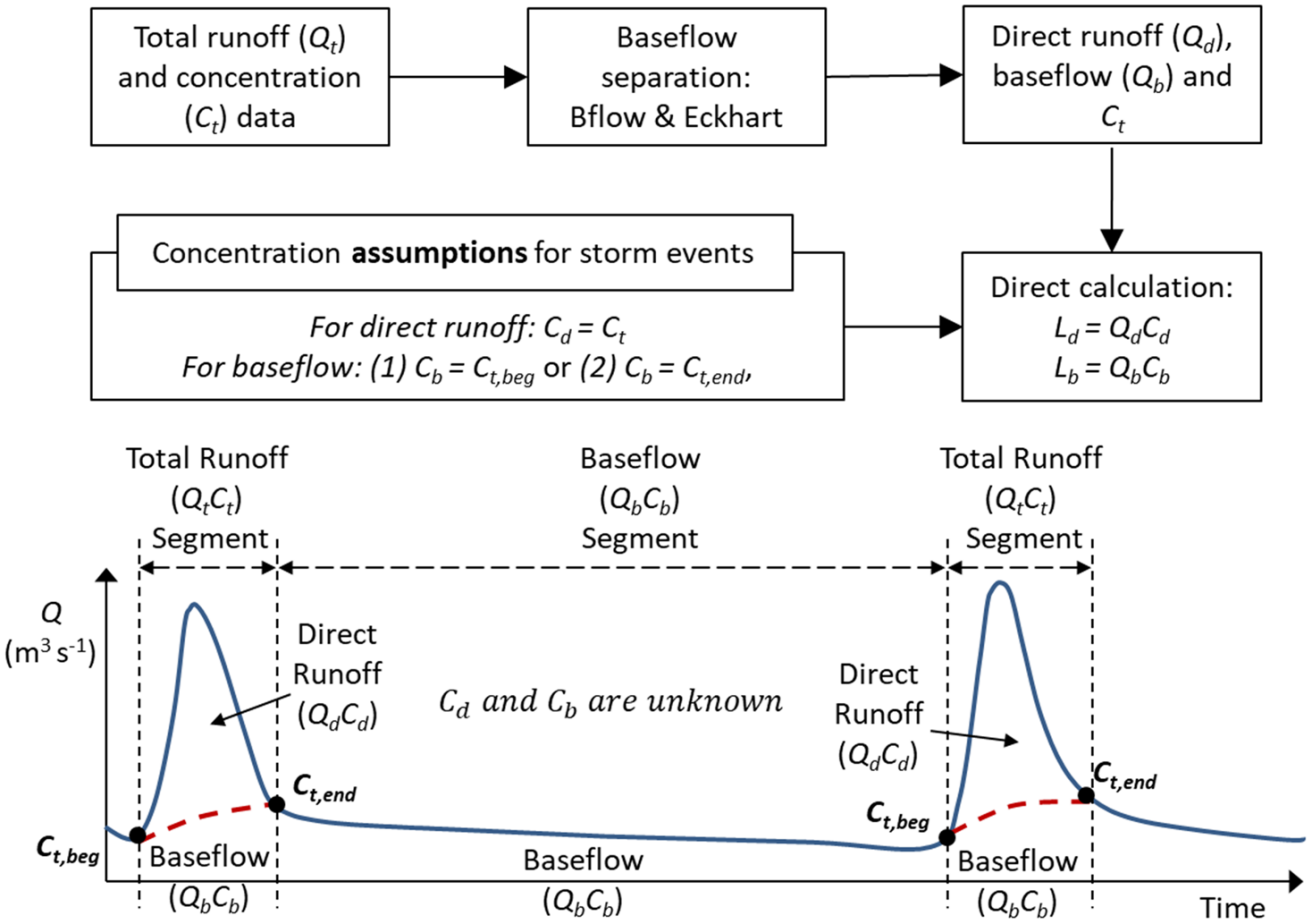
A framework proposed for the quantification of direct runoff and baseflow contribution to nitrate + nitrite loading. The ${C}_{t, beg}$ and ${C}_{t, end}$ represent the concentrations measured at the beginning or end of a storm event.

In this study, the contributions of direct runoff and baseflow to nitrate + nitrite loadings are quantified based on a mass balance relationship between total runoff, direct runoff, baseflow, and nitrate + nitrite concentrations associated with each of the hydrograph components (Fig. 2 and Table 2). When no direct runoff exists immediately or sometime after a storm event ends, the mass balance equation will become simple without any unknown variable because total runoff (hereafter runoff or streamflow) consists only of baseflow. In the case of a storm event (or during direct runoff exist in a streamflow hydrograph), the amount of direct runoff (${Q}_{d}$) and baseflow (${Q}_{b}$) can be obtained from total streamflow (${Q}_{t}$) using baseflow separation techniques (BFlow and Eckhardt; “Supporting text” section and Fig. S1 in the Supplementary Information). However, the mass balance relationship will have two unknown variables, the nitrate + nitrite concentrations of direct runoff (${C}_{d}$) and baseflow (${C}_{b}$) (Fig. 2 and Table 2). This study avoided the problem of such a “infinitely many solutions” by introducing assumptions developed based on hydrological reasonings about the temporal variation of baseflow and the streamflow composition within a storm event (or during direct runoff exists in the streamflow hydrograph).

**Table 2:** Assumptions for nitrate + nitrite concentrations in rainfall events and baseflow separation methods used in this study.

Case ID	Assumption	Baseflow separation method
Reference	None	Not applied
BB^a^	${C}_{b}={C}_{t, beginning}$ ${C}_{d}={C}_{t}$^b^	BFlow
TB	${C}_{b}={C}_{t, end}$ ${C}_{d}={C}_{t}$^b^	Bflow
BE	${C}_{b}={C}_{t, beginning}$ ${C}_{d}={C}_{t}$^b^	Eckhardt
TE	${C}_{b}={C}_{t, end}$ ${C}_{d}={C}_{t}$^b^	Eckhardt

Where ${C}_{t}$, ${C}_{d}$, and ${C}_{b}$ are the daily nitrate + nitrite concentrations of total runoff, direct runoff, and baseflow, respectively; ${C}_{t, beginning}$ and ${C}_{t, end}$ are the concentrations of total runoff at beginning and end of a rainfall event, respectively.

^a^The first and second characters of the case ID represent a concentration assumption and a baseflow separation method used, respectively.

^b^The nitrate + nitrite concentration of direct runoff (${C}_{d}$) was assumed the same as that of total runoff (${C}_{t}$) during rainfall events.

**Figure 3:**
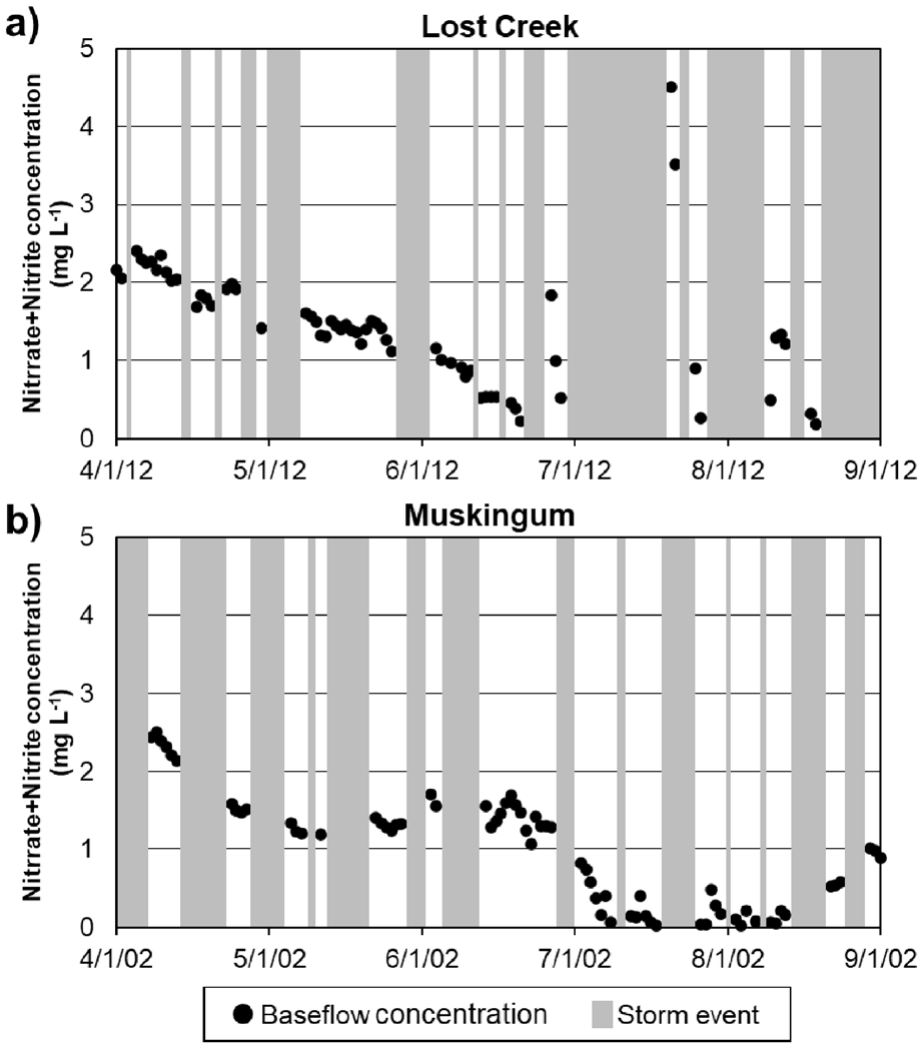
Temporal variations of baseflow nitrate + nitrite concentrations between rainfall events. (**a**) The Lost Creek watershed (with the smallest drainage areas) and (**b**) the Muskingum watershed (with the largest drainage areas).

In a rainfall event, streamflow is mainly provided by direct runoff. Thus, the nitrate + nitrite concentrations of streamflow discharges were assumed to be dominated by direct runoff, and in turn, ${C}_{d}$ can be approximated to ${C}_{t}$ during a rainfall event (Table 2 and Fig. 2). The nitrate + nitrite concentration of baseflow within a rainfall event may be different from that of direct runoff, but there is no known data that we can use to separate their concentrations. The time-series of baseflow’s nitrate + nitrite concentrations observed between rainfall events provided an idea that we could use to make reasonable assumptions about the variations of baseflow’s nitrate + nitrite concentrations (Fig. 3). Because baseflow discharge to streams does not change quickly^37,38^, we can assume that its nitrate + nitrite concentration also may not change rapidly within a rainfall event. In the smallest watershed (i.e., Lost Creek), for instance, baseflow nitrate + nitrite concentrations increased from 1.41 mg L^−1^ on 4/30/12, right before a rainfall event (50.0 mm) to 1.60 mg L^−1^ on 5/10/12 right after the event (Fig. 3a). On the other hand, the concentrations decreased from 2.04 mg L^−1^ on 4/13/12 to 1.69 mg L^−1^ on 4/17/12 after a rainfall event of 11.2 mm. In the case of the largest watershed (i.e., Muskingum; Fig. 3b), baseflow nitrate + nitrite concentrations decreased from 2.14 to 1.50 mg L^−1^ after a rainfall of 57.2 mm (4/13/12 to 4/25/12) but increased from 1.19 to 1.41 mg L^−1^ after another event of 67.8 mm (5/12/12 to 5/23/12). From the concentration data, we could not find mechanisms that can explain the different responses of baseflow nitrate + nitrite concentrations to rainfall events; however, we could see the differences in the baseflow nitrate + nitrite concentrations before and after rainfall events ranged from 0.17 to 3.31 mg L^−1^ (14 to 39% compared to the before- or after-event baseflow nitrate + nitrite concentrations) on average. Such a finding led us to making two assumptions: the nitrate + nitrite concentrations of baseflow would be close to the concentrations measured at (1) the beginning of a storm event (the cases BB and BE in Table 2) or (2) the end of the event (the cases TB and TE in Table 2). We believe the actual baseflow nitrate + nitrite concentrations may be between these two values (i.e., the concentrations at the beginning and end of an event); however, we decided to show the range (rather than average) and thus the uncertainty of nitrate + nitrite load contribution estimates in this study.

## Results

### Baseflow separation

**Figure 4:**
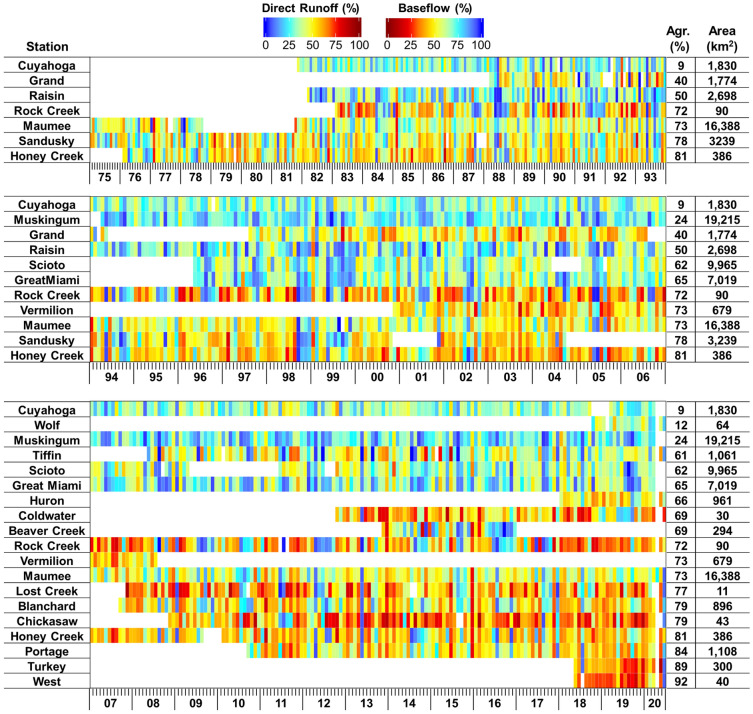
Monthly direct runoff and baseflow proportions in the 22 study watersheds (derived from the Bflow case). The x-axis represents years (last two digits of the years) and months (e.g., each interval between small lines means a month).

The baseflow separation analysis results showed that baseflow accounts for 33 to 72% of total runoff at the monitoring sites (or the watershed outlets) (Figs. 4, S2, and S3). The baseflow proportion to total runoff varied greatly by the drainage areas (Figs. 4 and S2). The statistical analysis showed that the baseflow and direct runoff proportions were correlated to the sizes of the drainage areas ($\left|r\right|$ > 0.70) and agricultural areas ($|r|$ > 0.75) across all the watersheds. The differences in the proportions of baseflow separated using the two methods (i.e., BFlow and Eckhart) were small, ranging from 0.1 to 4.0% (Fig. S3). In addition, no increasing or decreasing temporal trend was found in the baseflow and direct runoff proportions (Figs. 4 and S2). Streamflow was mainly comprised of direct runoff. For example, the interquartile range (IQR) of direct runoff proportions varied from 49% (25th percentile) to 73% (75th percentile) with the median of 62% when total runoff depths were greater than 10 mm, and the IQRs did not change substantially (e.g., IQR varied from 43 to 66% when the 100-mm runoff threshold was applied) while the runoff depth threshold changed from 10 to 100 mm.

### Nitrate + nitrite load estimation

**Figure 5:**
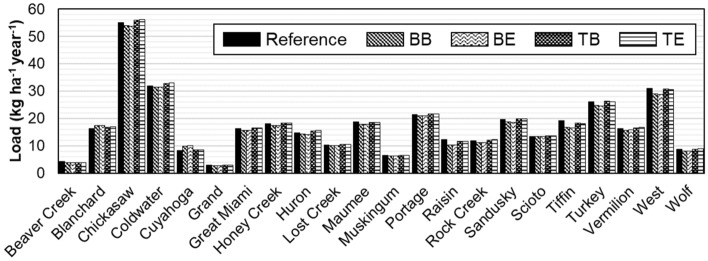
Comparison of annual average nitrate + nitrite loads estimated using the different methods (Tables 1 and 2).

The observed amount of nitrate + nitrite loads passing the monitoring stations (or the watershed outlets) ranged from 2.9 to 55.1 kg ha^−1^ year^−1^ (Fig. 5). There was no statistically significant trend detected in temporal changes in the annual loads at the 22 stations ($p>0.05$) (Fig. S4). A relatively small amount of load per unit area was found at Grand, Muskingum, and Cuyahoga presumably due to their relatively small sizes of agricultural areas. The annual loads were moderately correlated with the percentages of agricultural areas in the watersheds where drainage areas are larger than 100 km^2^ ($r$ = 0.73, $p<0.01$). The Chickasaw watershed was found to produce the largest amount of nitrate + nitrite loads per the unit area from prolonged high concentrations (10 to 55 mg/L) from December to June (Fig. S5). Intensive agricultural activities, including concentrated livestock operations, might be attributed to the relatively large amount of loads per the unit area of the Chickasaw and Coldwater watersheds^39^.

The nitrate + nitrite loads were relatively large in high-flow events from March to July. The summer months (May to August) generally receive a large amount of rainfall in a year^40^. In addition, the early-season discharge during March to May can occur by melted snowpack and high antecedent soil moisture conditions^7,8^. It is known that the March to July period strongly relates to the extent of algae bloom in the western basin^23,41^. Stow et al.^8^ showed that the early flow and load peaks during the critical season might reflect agricultural activities occurred during the preceding winter.

### Direct runoff and baseflow contribution to nitrogen loading

**Figure 6:**
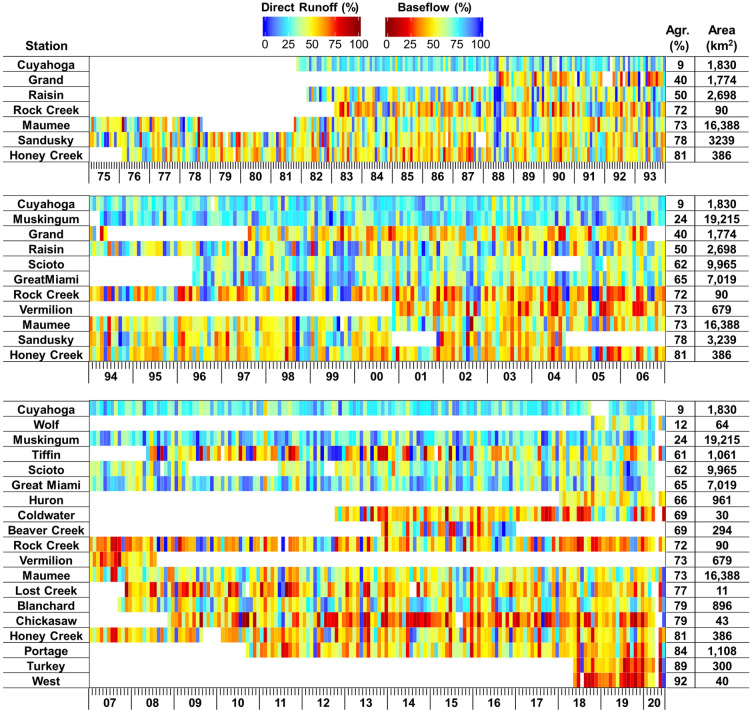
Monthly nitrogen loading contributions of direct runoff and baseflow in the 22 study watersheds (derived from the TB case). The x-axis represents years (last two digits of the years) and months (e.g., each interval between small lines means a month).

The analysis showed that baseflow accounts for 26 to 77% of the total nitrate + nitrite loadings at the monitoring sites, and the relative loading contributions of direct runoff and baseflow vary greatly by the drainage areas (Figs. 6, S6, and S7 and Table S1). Overall, the proportions of the relative nitrogen loading contributions of direct runoff and baseflow were changed seasonally within the observation periods (Figs. 6 and S6). In general, no temporal increasing or decreasing trend was found in the relative contribution proportions. In the case of Honey Creek, however, the nitrate + nitrite contributions of direct runoff seem to increase during the monitoring periods (*p* < 0.05). Such a finding implies land management changes (e.g., the expansion of tile drainage) toward increasing the drainage efficiency, especially in the agricultural drainage areas of the monitoring sites, one of the major sources of nitrogen^2,29^.

**Figure 7:**
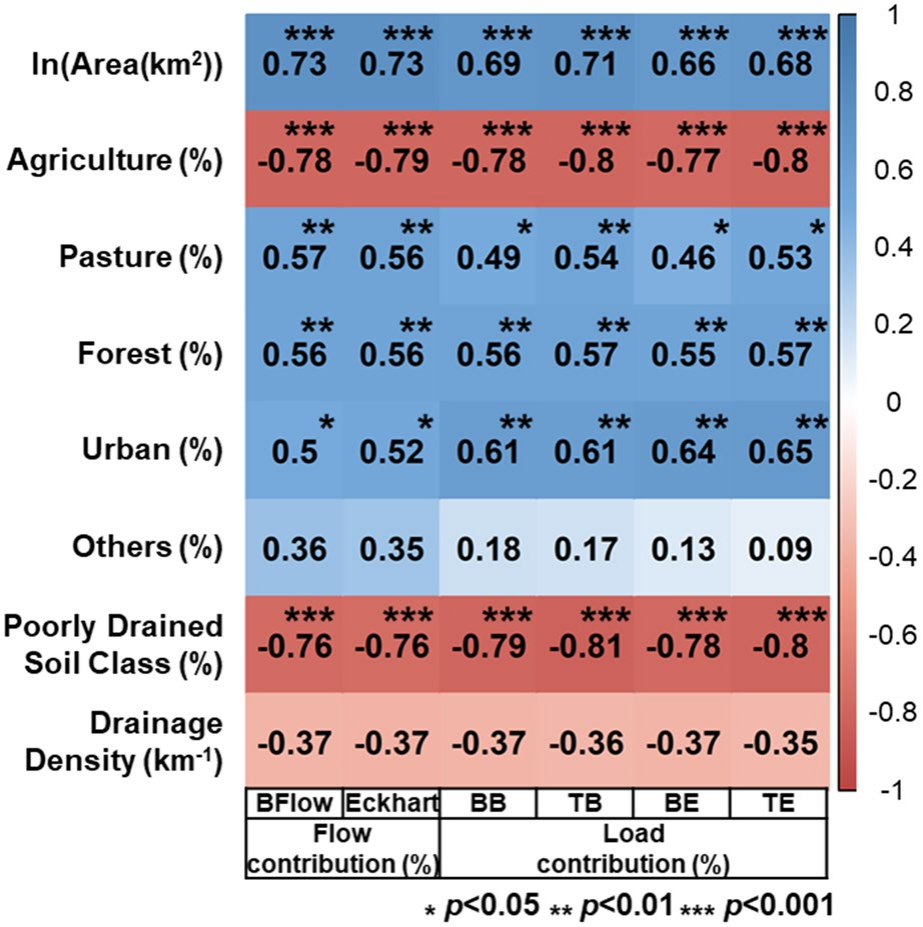
Correlation between baseflow contributions to nitrate + nitrite loading and selected watershed characteristics.

The relative contributions (%) of direct runoff and baseflow to nitrate + nitrite loading were compared by the stations (Figs. 6 and S6). In Figs. 6 and S6, the monitoring sites are listed in the order of the sizes of agricultural areas (km^2^) to identify their potential relationship with the contribution proportions. Overall, the baseflow contribution to the nitrate + nitrite loading decreased with increases in the sizes of agricultural areas. The correlation structure between the baseflow contributions and the land use compositions of the drainage watersheds was investigated to identify key factors (Fig. 7). The statistical analysis showed that the load contributions are moderately ($|r|$ > 0.6) associated with the sizes of the drainage areas and the percentages of agricultural area across all watersheds. The agricultural areas are relatively highly ($|r|>0.75$) correlated to the load contributions across all monitoring sites; all cases (BB, TB, BE, and TE) showed similar correlation coefficients (Fig. 7). The direct runoff contributions were strongly inversely correlated to the size of pasture, implying paster might reduce direct runoff and thus the amount of nitrate + nitrite loaded by direct runoff. The soil drainage levels (represented by the percentages of the poorly drained soils) were also significantly associated with the direct runoff (or baseflow) contributions ($|r|>0.75$), which might be attributed to the fact that the poorly drained soils in agricultural land are underlain by subsurface tile drains^2,18,33^. The stream network density might be a first-order control on hydrological response to precipitation^42^; however, it was not correlated to the nitrate + nitrite load contribution in this study. This result implies that tile drainage (or artificial subsurface drainage) rather than overland flow (or natural surface water drainage) might be the primary nitrate + nitrite loading pathway to streams in the study basins.

**Figure 8:**
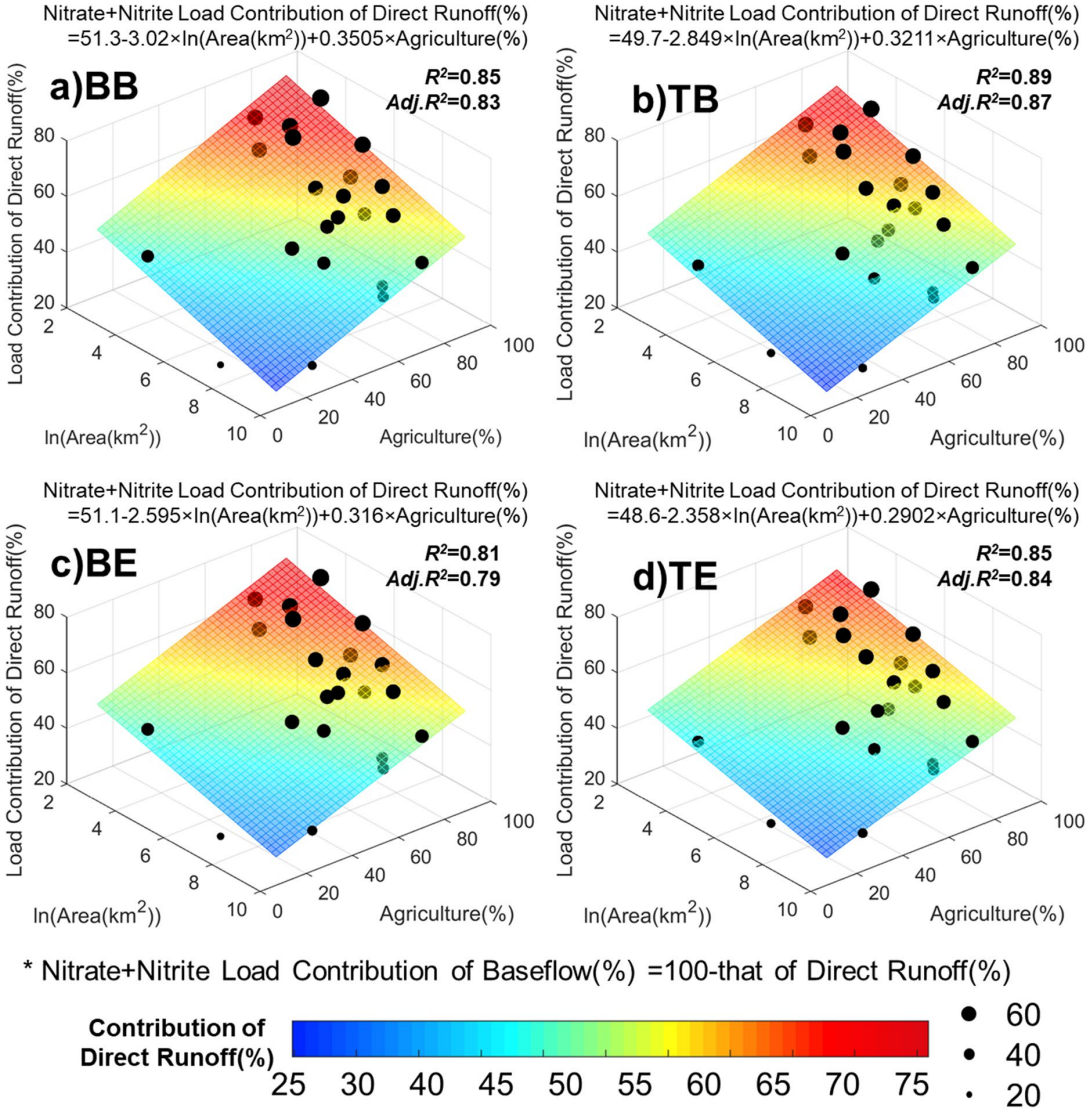
Relationship between the relative contributions of direct runoff to nitrate + nitrite loading, the sizes of drainage areas, and agricultural areas. (**a**) BB (*C*_*b*_ = *C*_*t,beginning*_ + BFlow), (**b**) TB (*C*_*b*_ = *C*_*t,end*_ + BFlow), (**c**) BE (*C*_*b*_ = *C*_*t,beginning*_ + Eckhardt), and (**d**) TE (*C*_*b*_ = *C*_*t,beginning*_ + Eckhardt).

Multiple linear regression was carried out to relate the nitrate + nitrite loading (dependent variables) to the watershed land use compositions and watershed characteristics (independent variables), which allows the development of a predictive model for the soluble nitrogen loads. Log-transformed drainage area sizes and the percentages of agricultural areas were selected as explanatory variables from the previous analysis (Figs. 7 and 8). In the regression analysis, all the cases provided adjusted ${R}^{2}$ greater than 0.75, but the use of BFlow for baseflow separation provided consistently higher ${R}^{2}$s compared to those of using the Eckhardt filter. The regression models of the four schemes were similar to each other (Fig. 8).

## Discussion

**Figure 9:**
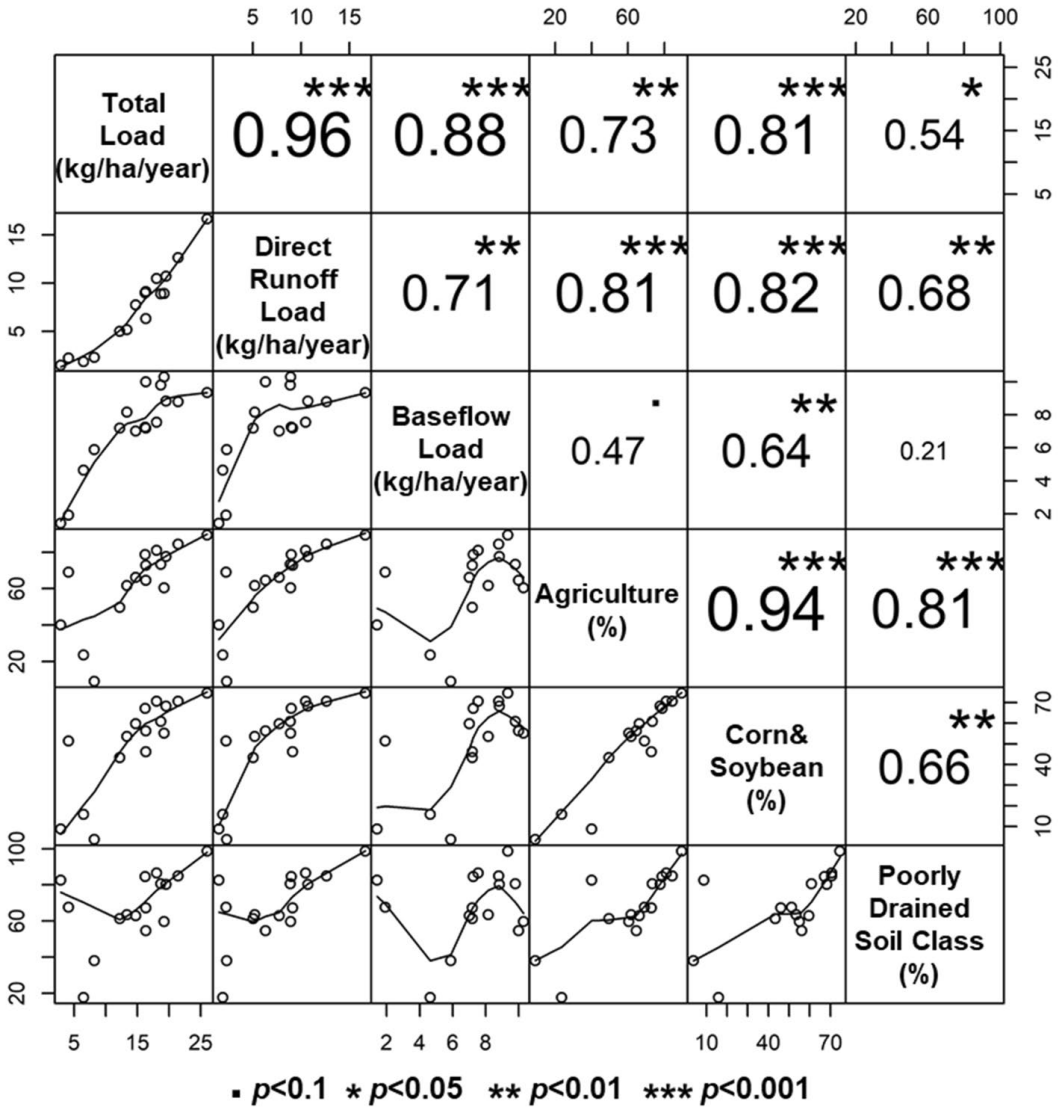
Correlation between nitrate + nitrite loads and agriculture and soil drainage characteristics (derived from the TB case). The percentages of areas with corn and soybean and poorly drained soil types (i.e., poorly drained, somewhat poorly drained, and very poorly drained) were determined using the USDA Cropland Data Layer (CDL) and USDA Soil Survey Geographic (SSURGO) database, respectively. The numbers shown on the axes represent ranges of variables in corresponding rows and columns.

The study results show that the extent of agricultural land use in given watersheds impact total and direct runoff loads (Fig. 9). The majority of cropped fields in the study watersheds are on poorly drained soils and intensively cultivated with corn (*Zea mays* L.) and soybean (*Glycine max* L.). The vast majority (> 80%) of agricultural fields were reported to have subsurface tile drainage systems to quickly drain excess water to ditches and streams from soils^19,23^. The results imply that the artificial drainage network in the study watersheds might be the primary nitrate + nitrite loading pathway to streams (Fig. 9). For instance, the amount of nitrate + nitrite loads contributed by direct runoff was significantly associated with the poorly drained soils and corn and soybean areas (*p* < 0.01). Such a result agrees with Williams et al.^19^ who showed that subsurface tile drains were key components driving nutrient transport processes during storm events in the western Lake Erie watersheds.

There are a few ways to isolate baseflow from direct runoff, and many of them employ digital filters that are not fully based on hydrological reasoning and concepts (Supplementary Information). Thus, the baseflow separation methods were reasonably suspected to be a source of uncertainty in the results of this analysis. However, the comparison between load estimates made using both filters showed that the nitrate + nitirite load estimates and the baseflow and direct runoff contribution estimates were not sensitive to the selection of the baseflow separation methods. Such a finding is supported by Zhu et al.^43^ who showed that there was no significant difference in the total nitrogen and phosphorus load estimates made using four different baseflow separation methods including the United Kingdom Institute of Hydrology method, the Lyne–Hollick method, the Eckhardt method, and the SWAT model.

This study roughly classified total runoff (or streamflow) into two components, direct runoff (fast runoff) and baseflow (slow runoff), according to the speed of their response to rainfall. In reality, there would be other water flow traveling at a variety of speeds. For example, direct runoff may include surface runoff and quick tile drainage, while baseflow may include groundwater flow, slow tile drainage, and bank storage return flow^19,44,45^. Yang et al.^45^ demonstrated that baseflow separated from streamflow (or total runoff) using different methods (e.g., the Eckhardt method) would include different flow components (e.g., slow interflow and bank storage return flow are included in baseflow separated using the Eckhardt method). In addition, they measured the conductivities of streamflow, direct runoff (or “rainfall runoff”), and baseflow and used them as a tracer to separate the baseflow (or direct runoff) from the streamflow. We used only flow discharge and nitrate + nitrite concentration data available for the study watersheds, which prevented this study from considering transient flow (e.g., interflow and bank storage return flow) in the analysis. Although the use of an environmental tracer can be expensive and laborious, it is expected to provide quality reference data that can help evaluate the reliability of baseflow separation methods and further understand water transport processes through the complex landscape^45^.

The assumptions about the nitrate + nitrite concentrations of baseflow and direct runoff within a storm event are another source of error and uncertainty. Schilling and Zhang^22^ assumed that the nutrient concentrations of baseflow would be the same as those of streamflow as long as the proportions of baseflow are equal to or greater than 90% of total runoff (or streamflow). Zhu et al.^43^ assumed the average nutrient concentrations of streamflow would be similar to those of baseflow during dry seasons (or winter in their study) presumably because the concentrations could be relatively stable compared to those in wet seasons (or summer) when rainfall events are relatively frequent (and thus non-point source pollutants are less likely to be transported). However, these and our studies could not explicitly consider the variations of the baseflow nutrient concentrations within rainfall events when baseflow as well as direct runoff increase due to the lack of data. The hydrological reasoning about the temporal variations of baseflow and streamflow composition helped develop assumptions about the nitrate + nitrite concentrations of baseflow during a storm event. The two assumptions (i.e., Table 2) did not create large differences in the nitrate + nitrite load and loading contribution estimates, suggesting that the assumptions of baseflow nitrate + nitrite concentrations might not be the major source of uncertainty in this study.

Although the nitrate + nitrite concentration assumptions did not add large uncertainty to the load estimates, they are not universally applicable. For instance, the ${C}_{d}={C}_{t}$ assumption was made based on the investigation of the relationship between flow and nitrate + nitrite concentrations (Figs. 3 and S8). We found that nitrate + nitrite concentrations did not change quickly before and after a rainfall event (Fig. 3), but the concentration changed substantially within an event (Fig. S8). We also found that streamflow was mainly comprised of direct runoff in a rainfall event (see “Baseflow separation” section). These findings agree with the previous studies that demonstrated the transport of nitrate or nitrite was mainly influenced by direct runoff during storm events^46–48^. However, streamflow may still contain baseflow even within a large rainfall event of a small watershed; thus, the amount of bias to be introduced by the assumptions into nitrate + nitrite load estimates may increase with increases in the baseflow contribution to the streamflow (or total runoff). In addition, our findings on the relationship between flow and nitrate + nitrite concentrations may not be true for other areas, depending on the hydrological conditions, including management practices, landscape and geological features.

The findings of the contribution of nitrogen loading pathways are expected to help advance the implementation plans and practices of agricultural conservation practices and guide the development of new practices for improved nitrogen load reduction efficiency^43,49,50^. The results indicate that the relative importance of direct runoff and baseflow vary spatially as a function of watershed sizes and land use compositions (the proportion of agricultural area) (Fig. 8). The relatively high direct runoff contributions were observed at the monitoring stations whose drainage areas are relatively small with the higher proportions of agricultural areas. Baseflow may increase with increases in drainage areas because of longer flow travel time that promotes the ‘reinfiltration’ of runoff on downstream areas^51–53^. Potential factors that control the nitrate + nitrite loading contribution of direct runoff from agricultural areas would be associated with agricultural management practices. For instance, excessive fertilizer application rates and improper application timing can promote fertilizer loss and agricultural nitrogen loading with rainfall events. In addition, surface crusting (or soil sealing) created by surface runoff and soil compaction can increase the proportion of direct runoff in agricultural fields^54,55^. Tile drainage, which is essential to efficient agricultural production in the Western Lake Erie watersheds with soils that have poor drainage capacity, can accelerate the soluble nitrogen leaching^17–19,26,56^. From a tile drainage monitoring study implemented in the Maumee watershed, Smith et al.^2^ found that tile discharge reached its peak at the same time when surface runoff was at its maximum discharge or even shortly before the surface runoff peak. Schilling and Helmers^57^ observed that peak tile discharge occurred even early in storm events. Such finding implies the significance of tile drainage and macropore flow in the nutrient loading in the Western Lake Erie areas, recommending advanced tile drainage management and implementation plans and practices^58^.

## Conclusion

This study quantified how much direct runoff and baseflow contributed to nitrate + nitrite loading to downstream water bodies in agricultural areas using baseflow separation methods and hydrological reasoning. In the 22 watersheds with water quality monitoring sites distributed in the Western Lake Erie areas, the nitrate + nitrite loading from the drainage areas ranged from 2.9 to 55.1 kg/ha/year depending on the sizes and land use compositions of the watersheds. The results showed that direct runoff was responsible for 23 to 74% of nitrate + nitrite loads. Baseflow was accountable to the other 26 to 77% of the loads. The relative nitrate + nitrite loading contributions of direct runoff and baseflow were significantly positively correlated with the proportions of agricultural areas and negatively correlated with the total drainage areas of the monitoring sites. The findings suggest that watershed management practices can be customized based on the watershed characteristics, such as sizes, cropping systems, and farming practices. The information of nitrate + nitrite load partitioning controlled by different watershed land cover configurations is expected to help better understand soluble nutrient loading mechanisms and develop targeted watershed management plans for improved downstream water quality.

## Supplementary Information

Supplementary Information.

## Data Availability

The water quality data used for this analysis are publicly available (at https://ncwqr.org/monitoring/data).
