# Supplementary material for: Quantifying the contribution of direct runoff and baseflow to nitrogen loading in the Western Lake Erie Basins
Source: Sci Rep. 2022 Jun 2;12:9216. doi: 10.1038/s41598-022-12740-1 (PMC9163129; doi:10.1038/s41598-022-12740-1)
Supplement: Supplementary file 1 — Supplementary Information. [file 41598_2022_12740_MOESM1_ESM.docx]

**Supplementary Information for:**

Quantifying the contribution of direct runoff and baseflow to nitrogen loading in the Western Lake Erie Basins

Jung-Hun Song ^1^, Younggu Her ^1*^, and Tian Guo ^2^

^1^ Department of Agricultural and Biological Engineering & Tropical Research and Education Center, University of Florida, Homestead, FL 33031, USA

^2^ Department of Agricultural and Biological Engineering, Purdue University

* Corresponding Author: Younggu Her, [yher@ufl.edu](mailto:yher@ufl.edu), 18905 SW 280^th^ St., Homestead, FL 33031, U.S.

**Contents of these files:**

1. **Supplementary text**
2. **Supplementary figures**
3. **Supplementary tables**

# Supplementary text

## 1.1 Baseflow separation

Total runoff was split into direct runoff and baseflow using baseflow separation techniques, and then the time series of each hydrograph component (direct runoff or baseflow) were used to quantify the amount of nitrate+nitrite transported with the component. Several techniques have been proposed to separate baseflow (or direct runoff) from total runoff or stream flow. Lyne and Hollick ^1^ and Arnold and Allen ^2^ extracted baseflow components from stream flow hydrographs based on the hydrological understanding of hydrograph partitioning. Their method is available with the BFlow software ^2^. Eckhart ^3^ applied an electronic signal filter to baseflow separation, and they proposed parameter values of the filter so that different geological features can be considered in the separation. Such separation techniques have been widely used in hydrologic analyses ^4–6^, and this study investigated the impacts of selecting baseflow separation methods on the results (Figure S3).

The BFlow method adopted the use of a two-parameter filter used in the signal analysis ^1^, assuming that baseflow is the low frequency component of total runoff ^2,7^. The equation of the filter is:

$b_{t}=\alpha b_{t-1}+\frac{(1-\alpha)}{2}(Q_{t}+Q_{t-1})$ subject to $b_{t}\ll Q_{t}$

where $b_{t}$ is the filtered baseflow at $t$ time step (day), $Q_{t}$ is the total runoff (day), and $\alpha$ is a filter parameter. The signal filtering method has no solid physical basis, but it is objective and reproducible ^2,8^. The application of the signal filter is limited to the condition where baseflow is relatively much smaller than total flow ($b_{t}\ll Q_{t}$) so as to avoid making an estimate of baseflow greater than that of streamflow ^3,7^. The BFlow method computes baseflow bypassing the filter over total runoff three times: forwards (1-Pass), backwards (2-Pass), and forwards again (3-Pass) ^7,9^. Baseflow estimates made using 1‐Pass was found consistent with the baseflow estimated with manual and technique within ±11% at an annual scale ^2^; thus, this study employed the 1‐Pass of the BFLOW method ^9^. The filter parameter ($\alpha)$ of the BFlow was reported to vary from 0.90 to 0.95, and the $\alpha$ value of 0.925 was selected for this study ^9,10^.

The Eckhardt filter is a two-parameter signal filter ^3^:

$b_{t}=\frac{\left( 1-{BFI}_{max} \right)ab_{t-1}+(1-a){BFI}_{max}Q_{t}}{1-a{BFI}_{max}}$ subject to $b_{t}\ll Q_{t}$

where $a$ is the baseflow recession constant (dimensionless) and ${BFI}_{max}$ is the maximum value of the long-term ratio of baseflow to total streamflow. This filter assumes that the groundwater system acts as a linear reservoir, which leads to baseflow recesses exponentially during dry-weather periods without groundwater recharge ^3^. The filter also limits the maximum ratio of baseflow to streamflow (${BFI}_{max}$), and the constraint can be potentially beneficial since the frequency spectrum of direct runoff has a broad bandwidth with a non-negligible low-frequency part ^11^. Eckhardt ^3^ suggested using the ${BFI}_{max}$ value of 0.80 for perennial streams with porous aquifers, 0.50 for ephemeral streams with porous aquifers, and 0.25 for perennial streams with hard rock aquifers; the value of 0.80 was used in this study. The baseflow recession constant ($a$) of the Eckhardt filter (or method) for Northern American watersheds ranges from 0.96 to 0.99, and $a$ value of 0.98 was selected for this study ^3,12^.

## 1.2 Uncertainty analysis

This study investigated the impact of the parameter value selection for the two baseflow separation methods on the contribution estimates. In the uncertainty analysis, the filter parameter of the BFlow method changed from 0.90 to 0.95 ^9,10^, and the baseflow recession constant of the Eckhardt method varied from 0.96 to 0.99 ^3,13^. The uncertainty analysis showed that the selection of parameter (the filter parameter of the BFlow method and the baseflow recession constant of the Eckhardt method) values did not substantially affect the nitrate+nitrite load contribution estimates (Fig. S7). For example, the baseflow nitrate+nitrite load contribution estimates varied by 1% to 11%, depending on the parameter value selection across the watersheds. The baseflow (or direct runoff) proportion estimates varied by 6% to 22%, depending on the parameter value selection (Fig. S3). The uncertainty (1% to 11%) in the baseflow nitrate+nitrite load contribution estimates was smaller than that (6% to 22%) of baseflow proportion estimates, which is attributed to the fact that the variations of nitrate+nitrite concentrations were much less than those of flow discharge. For instance, the coefficient of variation (CV) of nitrate+nitrite concentrations varied from 0.39 to 1.14 (with the average of 0.69) across all the 22 watersheds, while the CV of total runoff ranged from 0.94 to 4.02 (with the average of 2.01).

**References:**

1. Lyne, V. & Hollick, M. Stochastic time-variable rainfall-runoff modelling. in *Institute of Engineers Australia National Conference* vol. 1979 89–93 (Institute of Engineers Australia Barton, Australia, 1979).

2. Arnold, J. G. & Allen, P. M. Automated methods for estimating baseflow and ground water recharge from streamflow records. *J. Am. Water Resour. Assoc.* **35**, 411–424 (1999).

3. Eckhardt, K. How to construct recursive digital filters for baseflow separation. *Hydrol. Process.* **19**, 507–515 (2005).

4. Gonzales, A. L., Nonner, J., Heijkers, J. & Uhlenbrook, S. Comparison of different base flow separation methods in a lowland catchment. *Hydrol. Earth Syst. Sci.* **13**, 2055–2068 (2009).

5. Taormina, R., Chau, K.-W. & Sivakumar, B. Neural network river forecasting through baseflow separation and binary-coded swarm optimization. *J. Hydrol.* **529**, 1788–1797 (2015).

6. Tongal, H. & Booij, M. J. Simulation and forecasting of streamflows using machine learning models coupled with base flow separation. *J. Hydrol.* **564**, 266–282 (2018).

7. Partington, D. *et al.* Evaluation of outputs from automated baseflow separation methods against simulated baseflow from a physically based, surface water-groundwater flow model. *J. Hydrol.* **458–459**, 28–39 (2012).

8. Arnold, J. G., Allen, P. M., Muttiah, R. & Bernhardt, G. Automated base flow separation and recession analysis techniques. *Groundwater* **33**, 1010–1018 (1995).

9. Ahiablame, L., Chaubey, I., Engel, B., Cherkauer, K. & Merwade, V. Estimation of annual baseflow at ungauged sites in Indiana USA. *J. Hydrol.* **476**, 13–27 (2013).

10. Nathan, R. J. & McMahon, T. A. Evaluation of automated techniques for base flow and recession analyses. *Water Resour. Res.* **26**, 1465–1473 (1990).

11. Spongberg, M. E. Spectral analysis of base flow separation with digital filters. *Water Resour. Res.* **36**, 745–752 (2000).

12. Lim, K. J. *et al.* Automated Web GIS based hydrograph analysis tool, WHAT. *J. Am. Water Resour. Assoc.* **41**, 1407–1416 (2005).

13. Lim, K. J. *et al.* Development of genetic algorithm-based optimization module in WHAT system for hydrograph analysis and model application. *Comput. Geosci.* **36**, 936–944 (2010).

# Supplementary figures


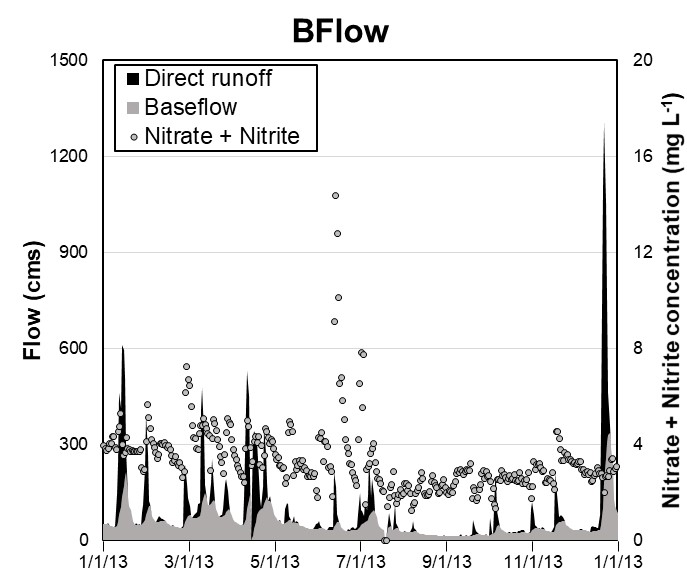


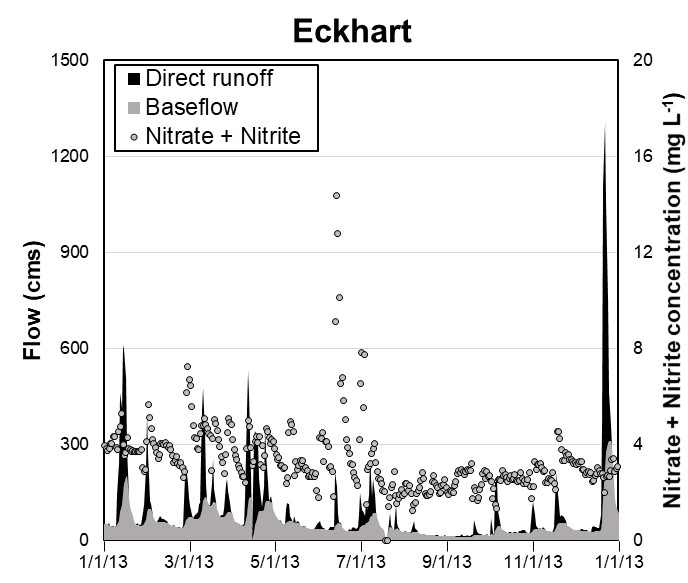


**Figure S1**. Examples of baseflow and direct runoff hydrographs separated from total runoff using the BFlow and Eckhart methods (the Great Miami watershed).


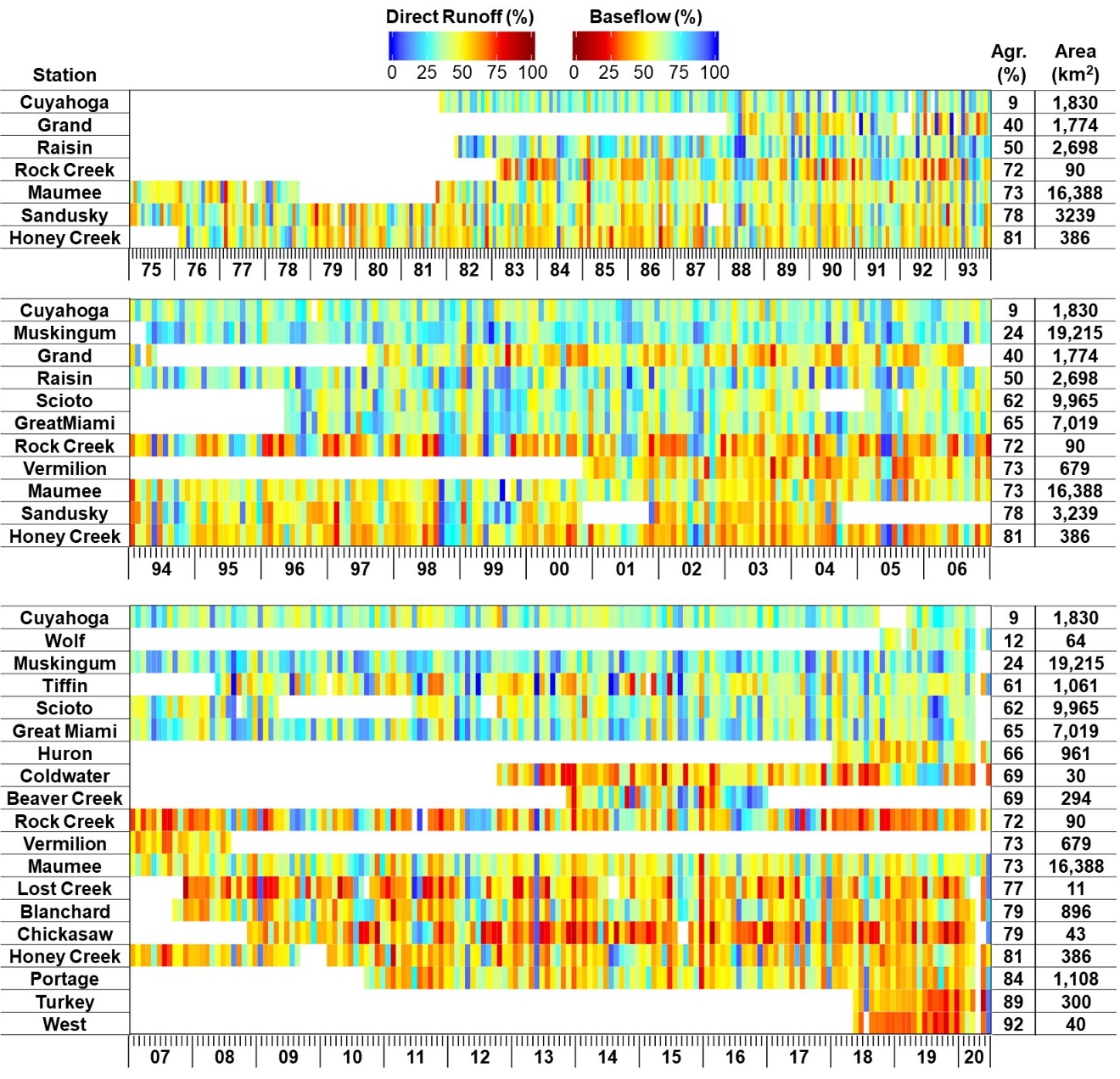


**Figure S2**. Monthly direct runoff and baseflow proportions in the 22 study watersheds (derived from the Eckhardt case). The x-axis represents years (last two digits of the years) and months (e.g., each interval between small lines means a month).


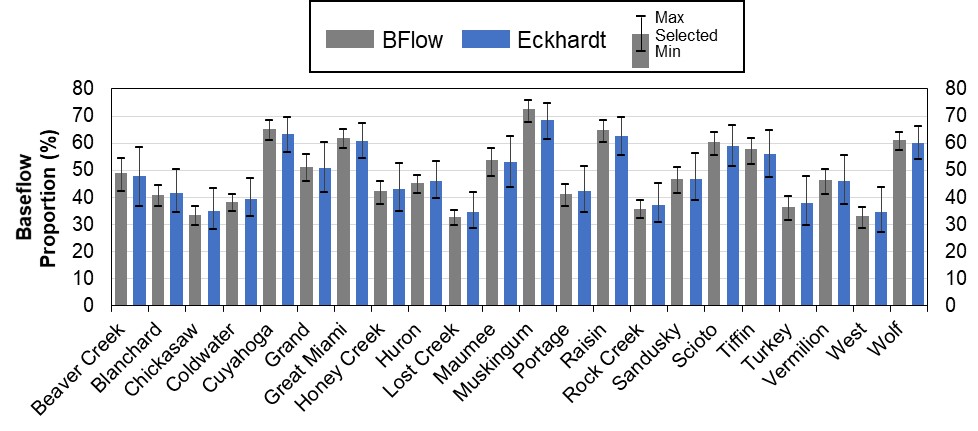


**Figure S3**. Variations of baseflow proportion estimates due to the selection of the filter parameter of the BFlow method and baseflow recession constant of the Eckhardt method.


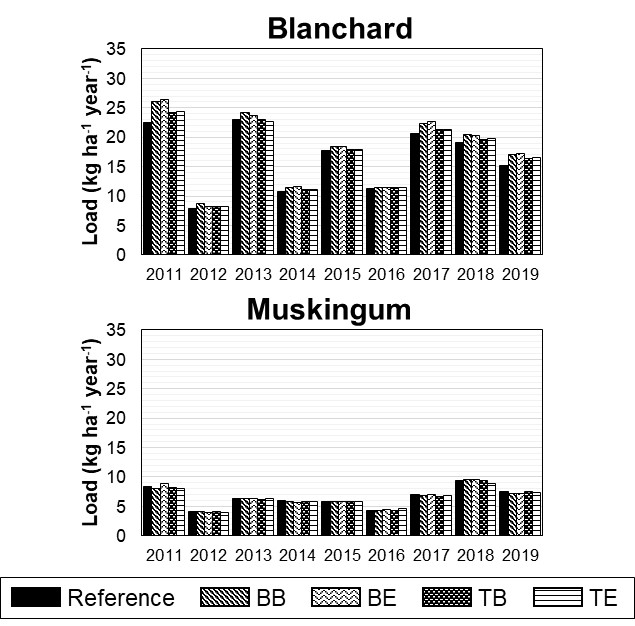


**Figure S4.** Annual variations in nitrate+nitrite loads estimated for the Blanchard and Muskingum watersheds.


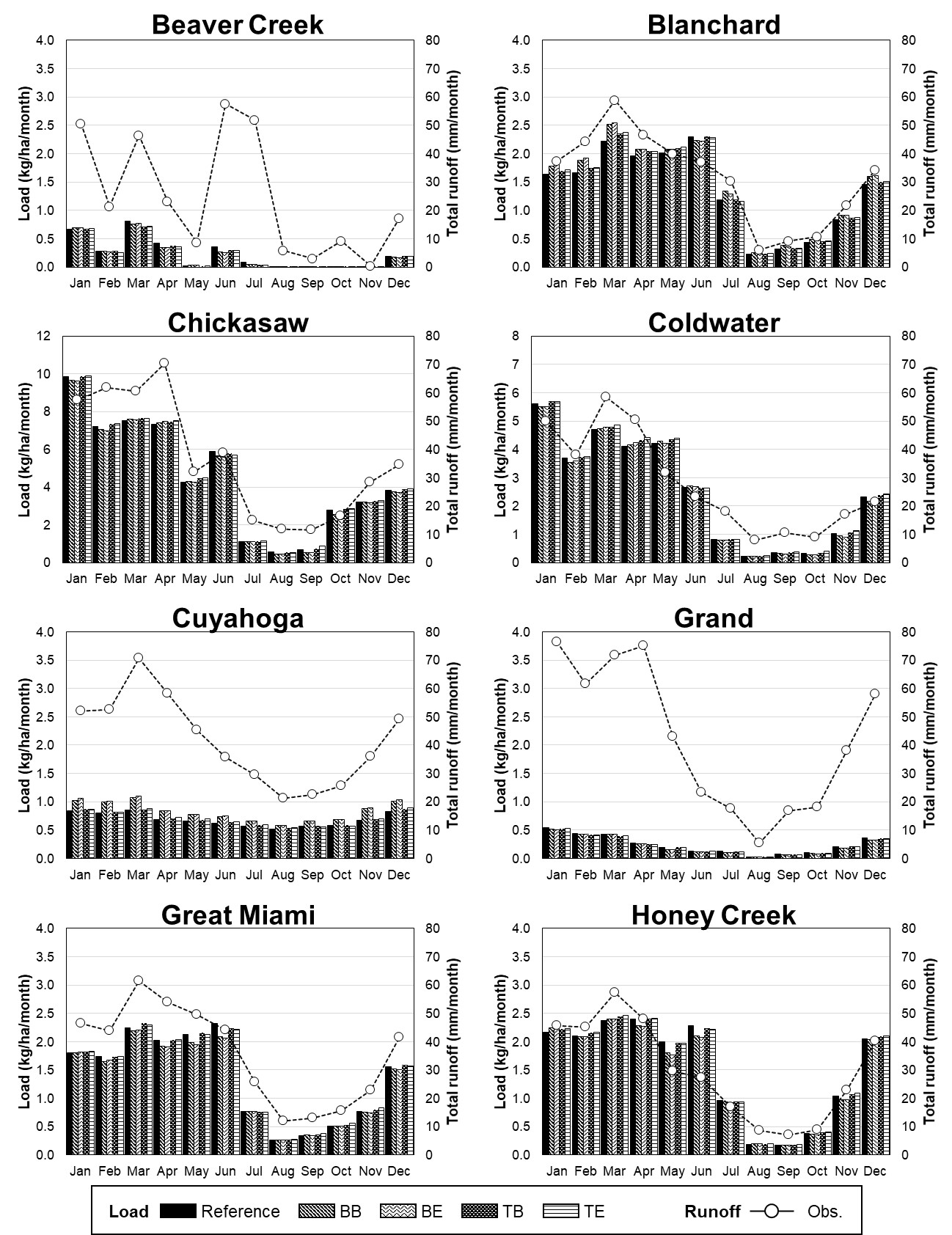


**Figure S5.** Monthly variations in nitrate+nitrite loads estimated for the individual watersheds.


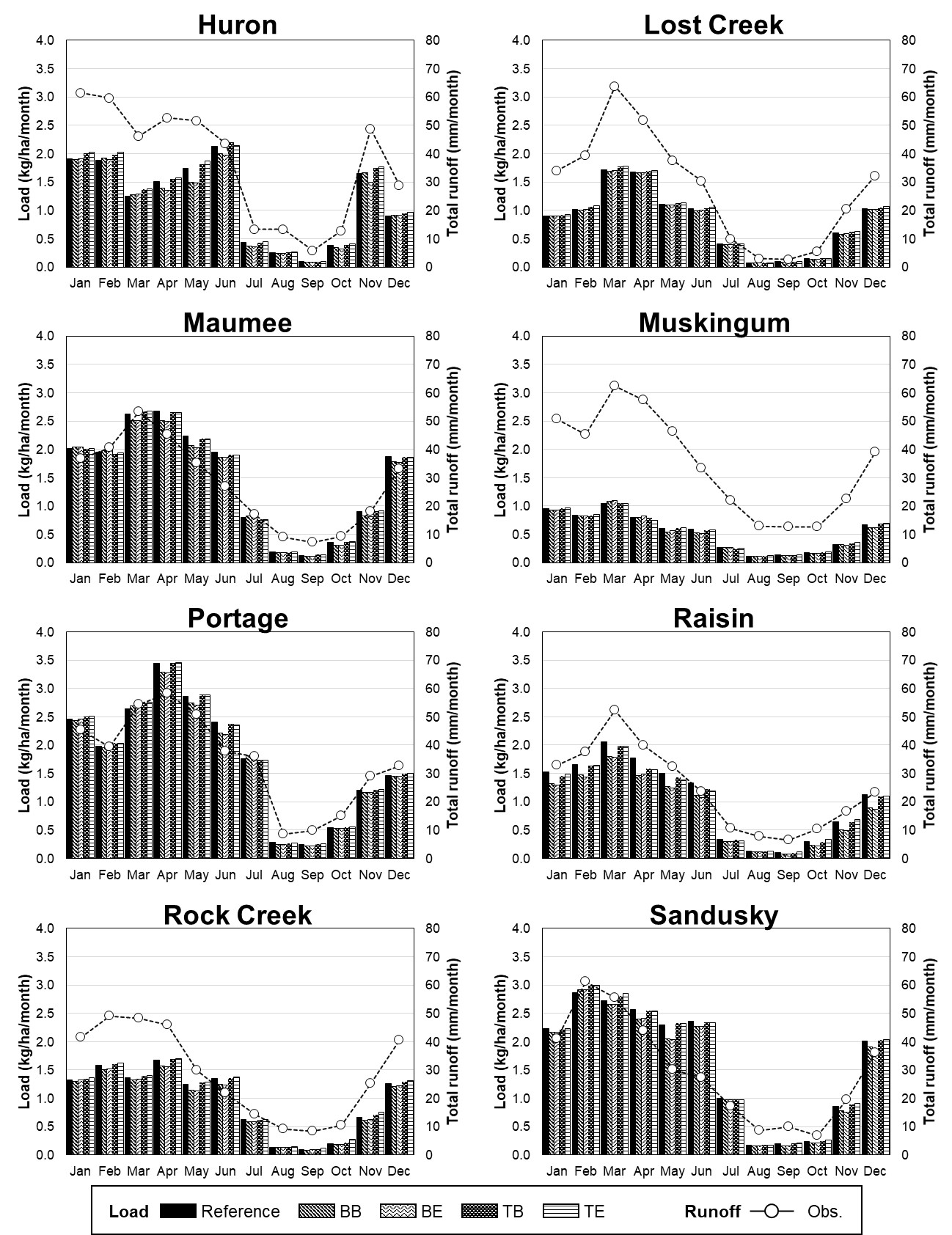


**Figure S5.** (Continued)


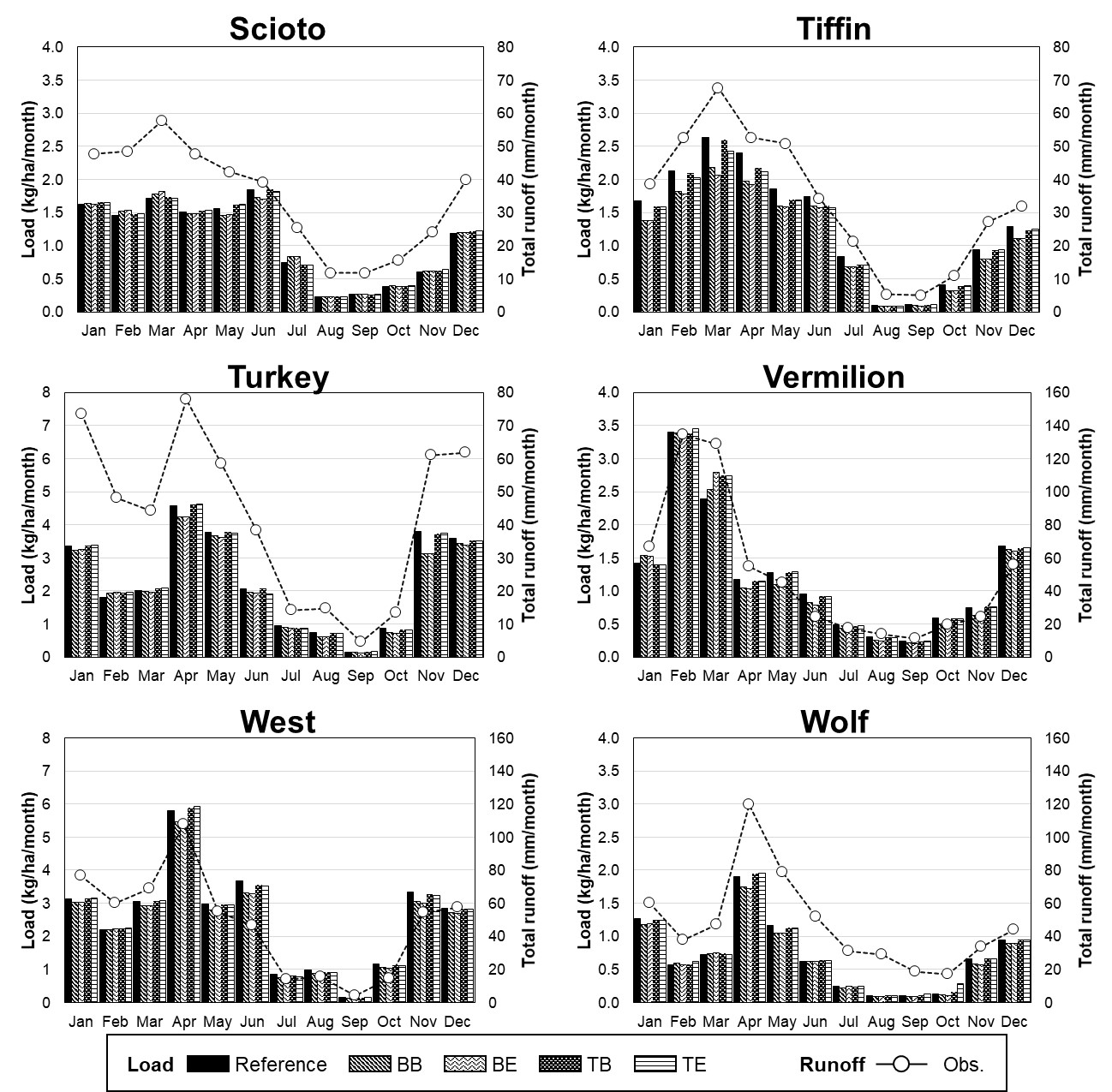


**Figure S5.** (Continued)


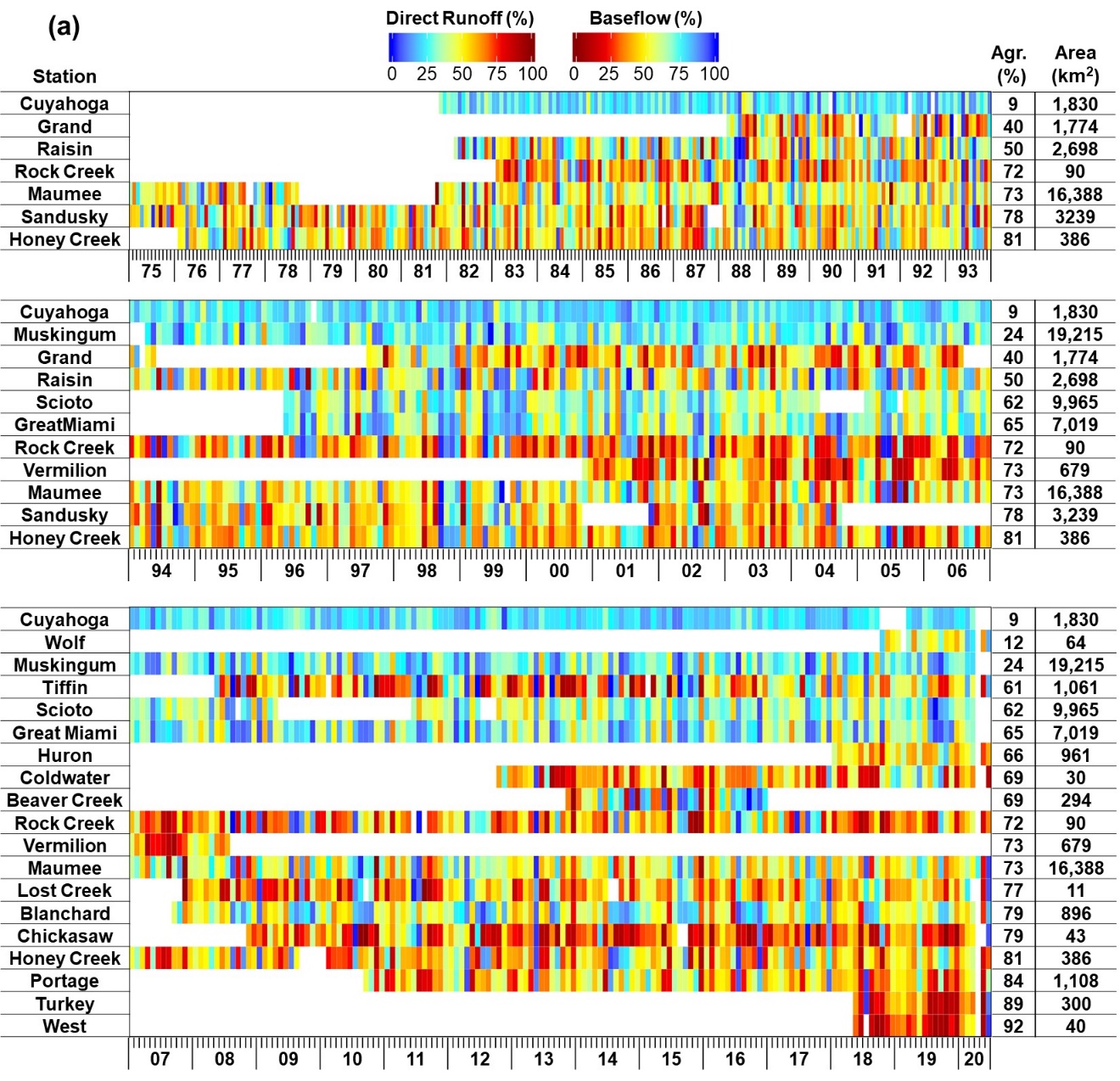


**Figure S6**. Monthly nitrogen loading contributions of direct runoff and baseflow in the 22 study watersheds: (a) BB, (b) BE, and (c) TE cases. The x-axis represents years (last two digits of the years) and months (e.g., each interval between small lines means a month).


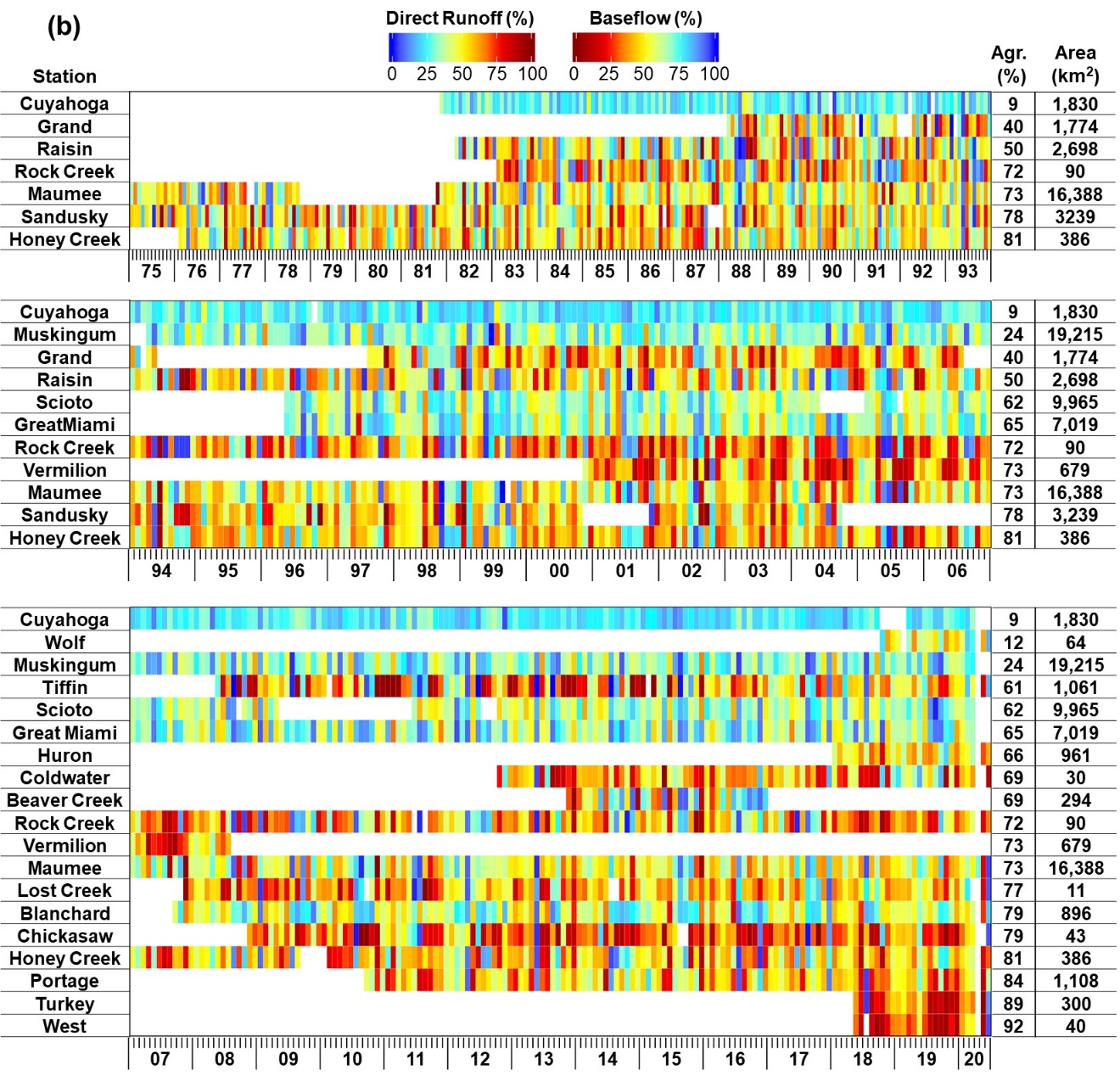


**Figure S6**. (Continued)


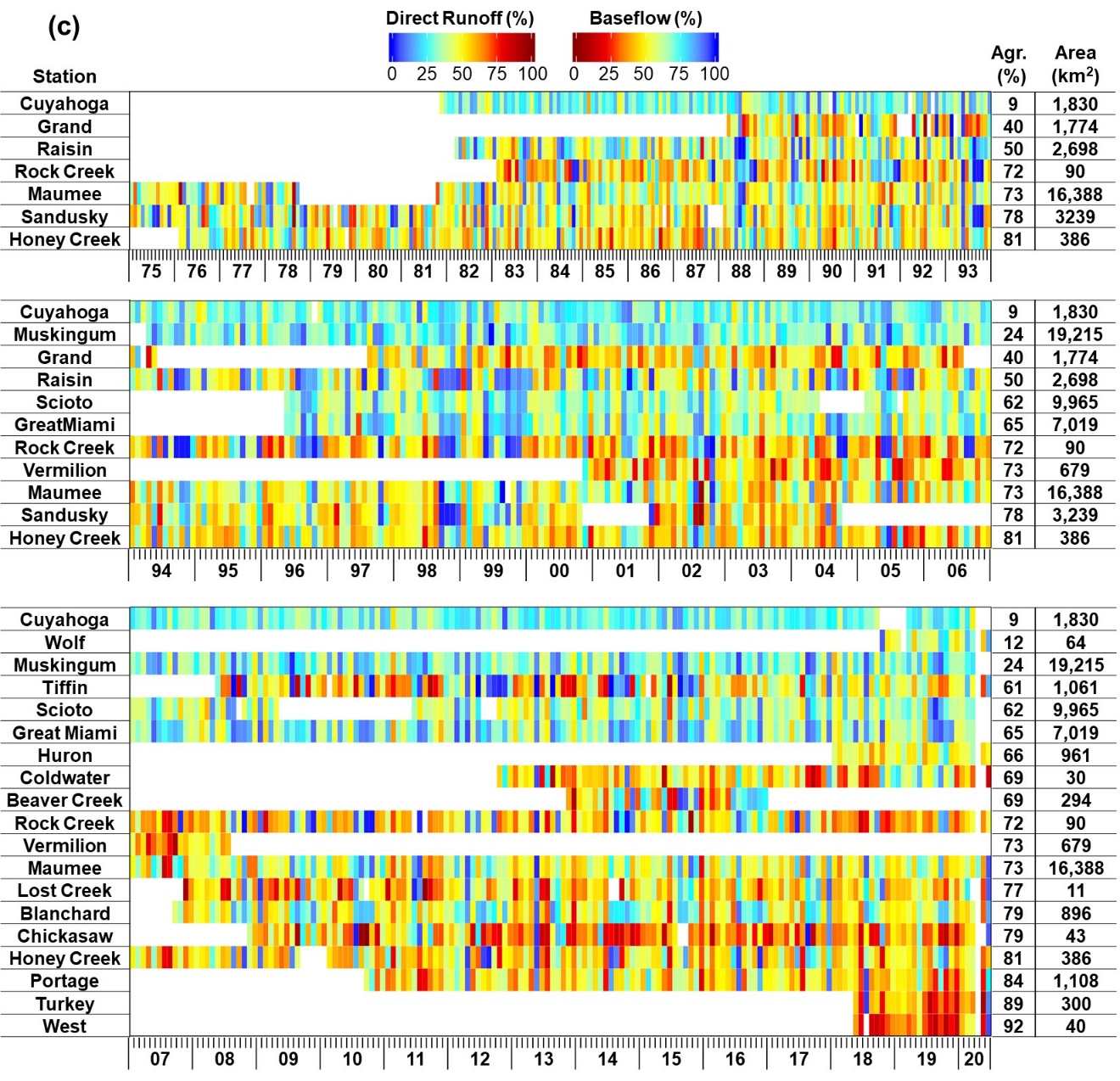


**Figure S6**. (Continued)


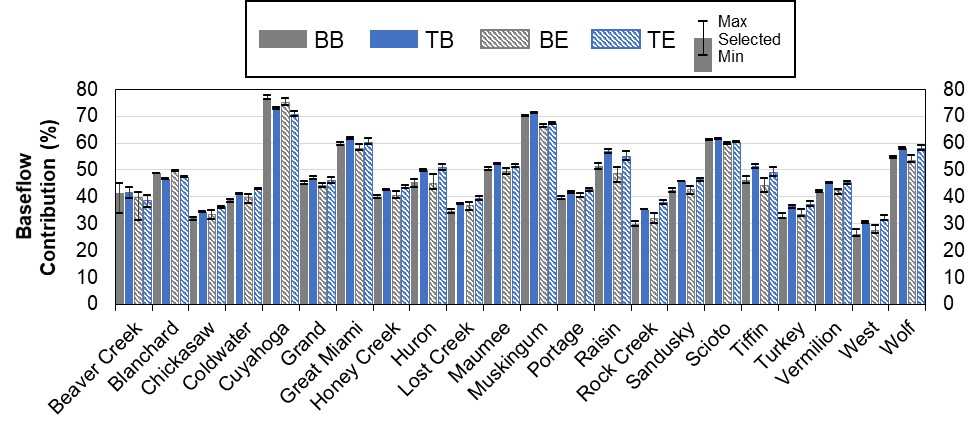


**Figure S7**. Variations of baseflow nitrate+nitrite load contribution estimates due to the selection of the filter parameter of the BFlow method and baseflow recession constant of the Eckhardt method.


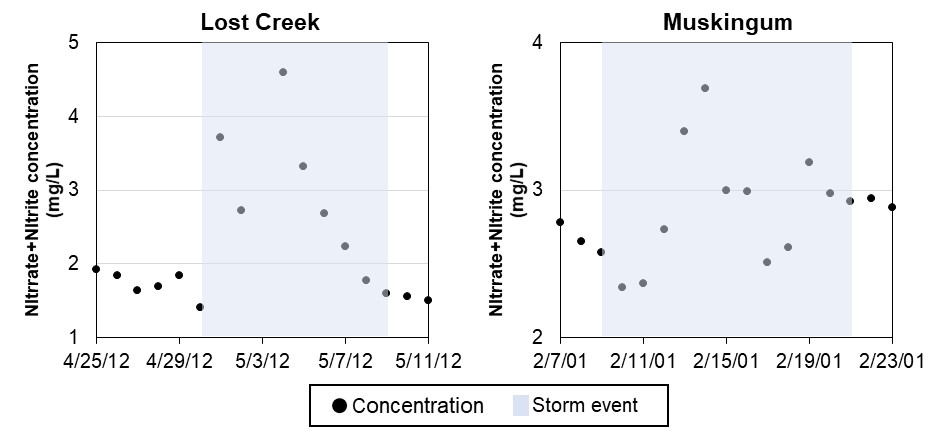


**Figure S8.** Temporal variations of nitrate+nitrite concentrations during rainfall events.

# Supplementary tables

**Table S1.** Nitrate+nitrite loading contributions of direct runoff and baseflow during the entire monitoring periods.

| Station | Percentage of nitrogen loading contributions | | | | | | | |  |
| --- | --- | --- | --- | --- | --- | --- | --- | --- | --- |
|  | BB | | TB | | BE | | TE | |  |
|  | DR^a^ | B^b^ | DR | B | DR | B | DR | B |  |
| Beaver Creek | 58 | 42 | 58 | 42 | 60 | 40 | 61 | 39 |  |
| Blanchard | 51 | 49 | 53 | 47 | 51 | 49 | 53 | 47 |  |
| Chickasaw | 68 | 32 | 66 | 34 | 67 | 33 | 64 | 36 |  |
| Coldwater | 61 | 39 | 59 | 41 | 60 | 40 | 57 | 43 | |
| Cuyahoga | 23 | 77 | 27 | 73 | 25 | 75 | 29 | 71 | |
| Grand | 55 | 45 | 53 | 47 | 56 | 44 | 54 | 46 | |
| Great Miami | 40 | 60 | 38 | 62 | 42 | 58 | 40 | 60 | |
| Honey Creek | 60 | 40 | 57 | 43 | 59 | 41 | 56 | 44 | |
| Huron | 55 | 45 | 50 | 50 | 55 | 45 | 49 | 51 | |
| Lost Creek | 65 | 35 | 63 | 37 | 63 | 37 | 61 | 39 | |
| Maumee | 50 | 50 | 48 | 52 | 51 | 49 | 49 | 51 | |
| Muskingum | 30 | 70 | 29 | 71 | 34 | 66 | 33 | 67 | |
| Portage | 60 | 40 | 58 | 42 | 60 | 40 | 57 | 43 | |
| Raisin | 49 | 51 | 43 | 57 | 52 | 48 | 45 | 55 | |
| Rock Creek | 70 | 30 | 65 | 35 | 68 | 32 | 62 | 38 | |
| Sandusky | 57 | 43 | 54 | 46 | 57 | 43 | 54 | 46 | |
| Scioto | 39 | 61 | 38 | 62 | 40 | 60 | 40 | 60 | |
| Tiffin | 54 | 46 | 49 | 51 | 56 | 44 | 51 | 49 | |
| Turkey | 68 | 32 | 63 | 37 | 67 | 33 | 63 | 37 | |
| Vermilion | 58 | 42 | 55 | 45 | 58 | 42 | 55 | 45 | |
| West | 74 | 26 | 70 | 30 | 72 | 28 | 68 | 32 | |
| Wolf | 45 | 55 | 42 | 58 | 46 | 54 | 42 | 58 | |

a: direct runoff; b: baseflow.
